# Assessing organizational implementation context in the education sector: confirmatory factor analysis of measures of implementation leadership, climate, and citizenship

**DOI:** 10.1186/s13012-017-0705-6

**Published:** 2018-01-08

**Authors:** Aaron R. Lyon, Clayton R. Cook, Eric C. Brown, Jill Locke, Chayna Davis, Mark Ehrhart, Gregory A. Aarons

**Affiliations:** 1 University of Washington, 6200 NE 74th Street, Suite 100, Seattle, WA 98115 USA; 2 University of Minnesota, 250 Education Sciences Bldg, 56 East River Road, Minneapolis, MN 55455 USA; 3 Department of Public Health Sciences, Miller School of Medicine, University of Miami, 1120 NW 14th Street, Office 104, Miami, FL 33136 USA; 4 University of Central Florida, 4111 Pictor Lane, Orlando, FL 32816-1390 USA; 5 University of California San Diego, 9500 Gilman Drive (0812), La Jolla, CA 92093 USA; 6 Child and Adolescent Services Research Center, 9500 Gilman Drive (0812), La Jolla, CA 92093 USA

**Keywords:** Implementation leadership, Implementation climate, Implementation citizenship, Schools, Education, Reliability, Structural validity

## Abstract

**Background:**

A substantial literature has established the role of the inner organizational setting on the implementation of evidence-based practices in community contexts, but very little of this research has been extended to the education sector, one of the most common settings for the delivery of mental and behavioral health services to children and adolescents. The current study examined the factor structure, psychometric properties, and interrelations of an adapted set of pragmatic organizational instruments measuring key aspects of the organizational implementation context in schools: (1) strategic implementation leadership, (2) strategic implementation climate, and (3) implementation citizenship behavior.

**Method:**

The Implementation Leadership Scale (ILS), Implementation Climate Scale (ICS), and Implementation Citizenship Behavior Scale (ICBS) were adapted by a research team that included the original scale authors and experts in the implementation of evidence-based practices in schools. These instruments were then administered to a geographically representative sample (*n* = 196) of school-based mental/behavioral health consultants to assess the reliability and structural validity via a series of confirmatory factor analyses.

**Results:**

Overall, the original factor structures for the ILS, ICS, and ICBS were confirmed in the current sample. The one exception was poor functioning of the Rewards subscale of the ICS, which was removed in the final ICS model. Correlations among the revised measures, evaluated as part of an overarching model of the organizational implementation context, indicated both unique and shared variance.

**Conclusions:**

The current analyses suggest strong applicability of the revised instruments to implementation of evidence-based mental and behavioral practices in the education sector. The one poorly functioning subscale (Rewards on the ICS) was attributed to typical educational policies that do not allow for individual financial incentives to personnel. Potential directions for future expansion, revision, and application of the instruments in schools are discussed.

## Background

A substantial body of implementation research has underscored the importance of organizational contexts in the successful adoption and sustainment of evidence-based practices (EBP) across various healthcare settings [1–5]. Findings indicate that even when high-quality implementation strategies––such as interactive training and post-training supports (e.g., observation and performance feedback)––are in place to facilitate professional behavior change, implementation outcomes are highly variable [6–8]. Additional research suggests that characteristics of the inner organizational setting, or the immediate context in which implementation occurs, have a substantial influence on the use of evidence-based practices in routine service delivery [9–11]. As a result, most leading implementation frameworks provide comprehensive coverage of “inner context” organizational factors [1, 3]. Conversely, inadequate attention to system influences is likely to cripple even the most well-resourced and thoughtful implementation efforts, leading some to observe that “bad systems trump good programs” [12]. However, there is a need for measures that capture key organizational context factors likely to set the stage for effective implementation. The current study examined the factor structure and psychometric properties of an adapted set of measures oriented to the inner organizational context for use in the education sector, an important service delivery context for delivery of behavioral health interventions and supports with considerable promise to positively impact public health of children and adolescents.

### Organizational implementation context

A wide variety of organizational characteristics have been identified as relevant to implementation, ranging from local policy to leadership and infrastructure [13]. These constructs vary, however, in the extent to which they are proximal and specific to the successful adoption and sustainment of EBPs. The *organizational implementation context* (OIC) reflects a subset of characteristics of the inner setting that are particularly relevant to the objective of EBP implementation. The OIC captures the factors within the immediate environment likely to influence front-line professionals’ EBP use. Conceptualized using the Exploration, Preparation, Implementation, and Sustainment (EPIS) [1] framework for implementation in public service systems, key OIC constructs include *strategic implementation leadership*, *strategic implementation climate*, and *implementation citizenship behavior*. These organizational constructs are considered focused or “strategic” in that they refer to specific organizational goals. This is in contrast to more general or “molar” versions of the construct (e.g., global organizational climate and culture, school climate) that, while important, provide a more comprehensive picture of the way an organization is functioning (e.g., general behavioral expectations at work, overall work stress) and are less directly linked to the strategic objective of EBP implementation [14].

#### Strategic implementation leadership

Strategic implementation leadership is a subcomponent of general leadership that involves specific behaviors that support or inhibit implementation in service organizations [15]. These include leaders being knowledgeable and able to articulate the importance of implementation and being supportive of staff, proactive in problem solving, and perseverant in the implementation process [15]. Importantly, strategic leadership exerts its strongest impact at an interactional level. Leaders who accomplish their strategic goals communicate regularly with staff, protect time during meetings to discuss strategic content, hold staff accountable, and provide ongoing feedback based on performance [16, 17]. In this way, strategic implementation leadership enhances the use of a number of “embedding mechanisms,” such as role modeling or setting clear criteria for rewards, [18] that communicate the importance of a strategic initiative and directly support staff use of new programs. Meta-analyses find that strategic leadership helps promote organizational change [19]. This finding is consistent with recent research on implementation strategies, which supports a link between enhanced implementation leadership and an organizational climate that is conducive to EBP implementation [20].

#### Strategic implementation climate

Defined as staff’s shared perception of the importance of EBP implementation [21], strategic implementation climate encompasses employee perceptions of the organizational supports and practices that help to define norms and expectations with regard to the implementation of new EBPs. A positive implementation climate signals what is expected, supported, and rewarded in relation to use of programs or practices [22]. Strategic implementation climate is supported by specific leadership behaviors that communicate those norms and expectations [15]. Similar to implementation leadership, strategic implementation climate reflects a subset of more general or molar organization climate, which is intended to reflect the entirety of the organizational setting. Existing research suggests that focused or strategic climates (e.g., safety climate) are most related to specific outcomes [23].

#### Implementation citizenship behavior

Citizenship behaviors are exhibited when employees go “above and beyond” their core job aspects or standard “call of duty” to further the mission of the organization [24]. Applying the concept to the goal of EBP adoption and sustainment, implementation citizenship behaviors are those that demonstrate a commitment to EBP by keeping informed about the EBP being implemented and supporting colleagues to meet EBP standards [25]. Because they represent actual changes in the behaviors of front line service providers, implementation citizenship behaviors mediate the influence of implementation leadership and implementation climate on implementation success [20].

### OIC assessment instruments

Instruments assessing the OIC constructs detailed above have been developed as part of a larger program of research focused on understanding and enhancing organizational factors that influence the implementation of mental and behavioral health programs and practices in public sector settings (e.g., community mental health, child welfare). Development of these measures has been largely consistent with the basic tenets of pragmatic measurement [26] in that they are low-burden (i.e., brief), sensitive to change, actionable (i.e., flowing into selection of implementation strategies [27]), and consistent with a larger framework or model (i.e., EPIS), among other criteria.

The implementation leadership scale (ILS) [15, 28] was developed to capture strategic leadership behaviors that likely drive successful EBP implementation***.*** The Implementation Climate Scale (ICS) [21] captures specific aspects of the inner organizational climate that are likely supportive of EBP implementation. Research has shown that the ICS correlates moderately with, but is distinct from, a conceptually similar strategic climate measure and correlates weakly with molar climate in mental health and child welfare settings. Last, the Implementation Citizenship Behavior Scale (ICBS) [25] assesses the degree to which providers within an organization go above and beyond their typical job roles and expectations to support EBP implementation. Together, these measures capture three critical factors associated with the OIC hypothesized to impact successful EBP implementation. Each instrument is described in more details in the “Method” section.

Although the strategic constructs assessed by the ILS, ICS, and ICBS are expected to be generalizable across settings, these measures are likely to require adaptation if they are to fit novel contexts of use (e.g., schools). Adaptation is often critical to improve the contextual appropriateness of instruments or practices [29]. Adaptation of measurement tools can include changes to existing items, terminology, and definitions to ensure that they are relevant and comprehensible to end users [30, 31]. Subsequent to those adaptations, studies should evaluate the extent to which the original factor structure is maintained to evaluate the validity of the tools in a new setting and provide information about their cross-setting utility. The current project was designed to conduct such an evaluation, following the adaptation of the ILS, ICS, and ICBS to support the implementation of mental and behavioral health programming in the education sector.

### OIC assessment in the education sector

Schools are the most common site for the delivery of behavioral health services to children and adolescents in the USA, a setting where upwards of 70–80% of service-connected youth receive care [32–36]. Consistent with the literature in the USA, we use “behavioral health” as an overarching term encompassing mental health and substance abuse services [37]. In schools, behavioral health includes a spectrum of services ranging from universal prevention to selected and indicated interventions [38]. A diverse school-based behavioral health workforce including educators (e.g., teachers) and dedicated healthcare personnel support this continuum of care [39].

School-based behavioral health consultants, who support systems and personnel to deliver evidence-based interventions across multiple levels of care, are frequently present in the education sector [38]. While they sometimes deliver direct services, these consultants often act as EBP champions (within school buildings) or intermediaries (across school buildings) to support implementation of behavioral health programs. Individuals functioning in this role are critical, given consistent evidence that school-based behavioral health services, while accessible, are unlikely to be evidence-based [39–41]. For instance, research suggests that, even when adopted, only 25–50% of school-based programs are implemented with acceptable fidelity, thus limiting their effects on student and school functioning [42].

In the education sector, the OIC reflects characteristics of inner organizational settings that impact implementation efforts, such as administrator and teacher norms and behaviors. Although prior research has focused on individual-level factors for reasons of convenience and feasibility, multilevel assessment is needed to successfully address implementation issues and install new programs [43]. Existing research in education has tended to focus narrowly on measuring implementation outcomes such as fidelity [44], with minimal attention to capturing the organizational factors that specifically impact delivery of EBPs. Careful assessment of the OIC in schools should consider multiple system levels and include perspectives of individual teachers and administrators, as well as organizational processes at the school and district levels [38].

Educational researchers have previously proposed strong principal leadership as a requirement for adoption and use of SEL programs [45] and examined leadership qualities as important predictors of school climate and school improvement [46, 47], but no studies have investigated strategic implementation leadership, climate, and citizenship. Although there are a number of general principal leadership measures with good psychometric properties [48], such as the Vanderbilt Assessment of Leadership in Education [49] and the Principal Instructional Management Rating Scale (PIMRS; [50, 51], existing measures in the educational sector remain too broad to identify specific leadership behaviors that are directed at EBP adoption, delivery, and sustainment in schools because they assess a diffuse range of general leadership qualities (e.g., transformational leadership). Similarly, educational researchers have long examined the role of school climate––defined as people’s perceptions of social norms, goals, values, interpersonal relationships, teaching and learning practices, and organizational structures [52]––and its connection to wellbeing and positive outcomes among educators and students. This has led to the development and use of a range of broad school climate measures (e.g., [53]) that, while useful in assessing organizational health, do not capture specific barriers impeding implementation or strategic implementation climate. As a result, these instruments lack utility regarding the selection of tailored implementation strategies to facilitate improved EBP use. Further, there is some educational research that has investigated broad (i.e., non-strategic) citizenship behavior among educators and found it to be related to their participation in decision-making, sense of self-efficacy, and perceived status in the organization [54, 55]. However, the concept of citizenship has not yet been specifically applied to the extent to which educators go beyond the “call of duty” to keep informed about EBPs and support their fellow colleagues to deliver EBP with fidelity [56]).

Despite their strong theoretical and empirical links to EBP implementation, none of the EPIS constructs have been studied systematically in schools, where successful implementation of universal EBPs can facilitate improvements in teacher behavior (e.g., instructional practices, interactions with students, use of reinforcement, etc.) that result in positive student outcomes [57]. As touched on above, in the education sector, existing measures of organizational processes have one or more of the following limitations: (1) they are most often either molar in nature (rather than specific to implementation) or intended for use with specific EBPs and not generalizable; (2) they lack an underlying theoretical framework; or (3) they do not translate to specific, practical actions that strategically improve implementation. Nevertheless, it is possible that the broad educational literature has conceptualized implementation in ways that are not readily identifiable using contemporary implementation science constructs. Finally, although the EPIS framework suggests that leadership, climate, and citizenship are inter-related aspects of the organizational context, education sector behavioral health research typically has explored them independently. This reflects a missed opportunity to understand the associations among these factors and the extent to which they may be complementary in promoting EBP use.

### Study aims

The current study aims were twofold. First, consistent with the original authors’ call to examine the utility of the ILS, ICS, and ICBS in other applied contexts [15], we sought to evaluate the construct validity of adapted versions of these three pragmatic measures through large-scale administration to school-based behavioral health consultants. As indicated above, behavioral health consultants were selected given their frequently central role in EBP implementation. Additionally, it provided an opportunity to explore the inter-relationships among those factors to determine shared and unique variance. Through this, it provided an opportunity to confirm the relevance of the strategic implementation leadership, climate, and citizenship constructs in schools as well as assess potential utility of the measures themselves.

## Method

### Participants

The sample included members from a state-wide, government-sponsored initiative on the west coast of the USA focused on the delivery of EBP to address youth behavioral health problems. Members were nominated by directors of regional special education agencies based on their commitment to engage in consultative efforts within their school systems. The regional agencies provide coverage of all the geographical areas in the state and are inclusive of school systems operating in rural, suburban, and urban environments. Membership is maintained through participation in annual forums that focus on implementation of EBP and ongoing state-wide research and evaluation activities. The organization was established approximately 20 years ago following federal and state legislation calling for educators to implement individualized behavior intervention plans for youth and has since evolved to focus more broadly on dissemination and implementation activities involving a continuum of universal, targeted, and intensive supports (i.e., multi-tiered systems of support [38]).

A total of 196 out of 212 total members participated (92%) and were included in analyses. Eighty percent of the respondents identified as female and 20% as male. Most respondents (76%) identified their race/ethnicity as White/non-Hispanic, followed by 11% Hispanic or Latino, 6% Black or African American, 5% Asian, 2% American Indian or Alaska Native, 2% Other, and 1% Native Hawaiian or Pacific Islander. Four percent of respondents elected not to disclose race/ethnicity information. The most commonly held highest-degree-earned was a Master’s degree (77% of respondents), with the remaining respondents holding an Educational Specialist (EdS) degree (10%), PhD (4%), PsyD (3%), Bachelor’s (1%), and “Other” (4%). Within the Other degree category, most reported holding board certification as a behavior analyst. Two percent of respondents were between 20 and 29 years of age, 35% were 30–39, 30% were 40–49, 23% were 50–59, and 7% were 60–69. The average number of years in their current profession was 15.8 (SD = 8.3 years). Due to missing data (< 5% overall), the number of participants included in some analyses was less than 196. Complete demographic information for participants is shown in Table 1.

**Table 1:** Demographics of survey respondents

Characteristic	Number	Percent
Gender		
Male	39	19.9
Female	155	79.1
Ethnicity		
American Indian or Alaska Native	4	2.0
Asian	10	5.1
Black or African American	12	6.1
Hispanic or Latino	22	11.2
Native Hawaiian or Pacific Islander	2	1.0
White/non-Hispanic	148	75.5
Other	4	2.0
Prefer not to disclose	7	3.6
Highest degree earned		
BA/BS	1	0.5
MA	151	77.0
EdS	19	9.7
PsyD	6	3.1
PhD	8	4.1
Other	8	4.1
Age		
20–29	4	2.0
30–39	68	34.7
40–49	58	29.6
50–59	45	23.0
60–69	14	7.1

### Procedures

Data were collected via an online survey, distributed through email. Prior to constructing the survey, researchers with expertise in school-based implementation adapted ILS, ICS, and ICBS items for the education context in collaboration with the developers of the original measures. Adaptations consisted of changing item wording to ensure construct equivalence for the target respondents (i.e., school-based practitioners [58]). An effort was made to preserve the integrity of the original items and constructs while ensuring appropriateness to the school context [59]. Thus, all items from the original scales were maintained with changes only made to item wording, such as replacing the word “supervisor” with “school administrator,” “clinician” with “school personnel,” and “agency” with “school.”

At the beginning of October 2015, members were sent an e-mail asking them to participate in an online survey examining their perceptions of the implementation of EBP to prevent or remediate student social, emotional, and behavioral problems. Weekly email reminders were sent for a period of up to 1 month to recruit as many respondents as possible. This University of Washington’s Human Subjects Institutional Review Board determined the study was exempt. As a result, no consent forms were collected, but information disclosures were presented to all participants prior to their completion of the online survey. Approval also was obtained by leadership from the participating state-wide organization. Researchers collaborated with the organization’s leadership surrounding the timing and administration of the web-based survey.

The current study was part of a larger project examining behavioral health consultants’ perceptions of the implementation of school-based EBPs and employed best practices in designing a web-based survey (e.g., visual ease, clear instructions, sending the survey, reminders, etc. [60]). The survey was divided in two sections: (a) respondents’ perceptions of organizational factors impacting uptake and use of universal school-based EBP and (b) respondents’ perceptions of facilitators and barriers to consulting with teachers to implement individualized EBP for youth with behavioral health problems. For this study, only the items from the first section were utilized. Given the importance of assessing respondents’ exposure to a school’s organizational context for the current assessment project, the survey included explicit instructions for respondents to consider and report on the school in which they spent the most time.

### Measures

As described above, all three of the OIC measures detailed below were adapted to ensure item wording was appropriate and relevant to the school context.

#### ILS

The original ILS was developed to assess the degree to which leaders engage in specific behaviors that are supportive of EBP implementation. All ILS items are scored on a five-point, 0 (“not at all”) to 4 (“very great extent”) scale. In previous work, exploratory factor analysis (EFA) resulted in a 12-item scale with four subscales representing proactive leadership, knowledgeable leadership, supportive leadership, and perseverant leadership. Subscale internal consistencies range from 0.95 to 0.98 [15]. Previous confirmatory factor analysis (CFA) supported the fit of the measurement model to the data and indicated a higher-order implementation leadership factor with all sub-factors beneath it. The ILS has demonstrated adequate internal consistency reliability as well as convergent and discriminant validity from related scales [15].

#### ICS

The original ICS was developed to assess the degree to which there is a strategic organizational climate supportive of EBP implementation. Thirty-eight items were developed and evaluated based on the development process described above. All ICS items are scored on a five-point, 0 (“not at all”) to 4 (“very great extent”) scale. Previous EFAs resulted in a final factor structure of six ICS dimensions: focus on EBP, educational support for EBP, recognition for EBP, rewards for EBP, selection for EBP, and selection for openness, with subscale internal consistency ranging from .81 to .91. In the original development studies, ICS items were reduced from 38 to 18, with three items falling under each factor. Confirmatory factor analyses supported the factor structure and additional analyses provided evidence supporting the reliability and construct validity for the ICS [21].

#### ICBS

The ICBS [25] was developed originally to assess the extent to which providers exceed typical expectations of their job to go above and beyond to support the implementation of EBPs. Initially, 10 items were developed and evaluated based on the development process described above. In the original measure, supervisors assessed each of their providers’ implementation citizenship behavior. All ICBS items are scored on a five-point, 0 (“not at all”) to 4 (“great extent”), scale. EFAs supported the proposed two-factor structure of the Implementation Citizenship Behavior Scale (ICBS): helping others and keeping informed. Items were reduced from 10 to 6, with three items falling under each factor. Internal consistencies range from .91 to .93. CFAs supported the factor structure and additional analyses provided evidence supporting the reliability and construct validity for the ICBS.

### Data analytic approach

To assess the construct validity of previously identified measurement models for the adapted scales, we conducted a series of confirmatory factor analyses (CFA) using weighted least squares means and variances (WLSMV) estimation with delta parameterization for the ordered-categorical scale items [61], as employed in M*plus* [62]. WLSMV estimation allows for missing item data under the assumption that the data are missing at random. Model fit was assessed based on a preponderance of the evidence from the chi-square statistic, comparative fit index (CFI; [63, 64]), the Tucker-Lewis index (TLI; [65]) and root mean square error of approximation (RMSEA; [66]) with values of the CFI and TLI greater than .95 and values of the RMSEA less than or equal to .05 as indicative of good model fit to the data. Based on Tabachnick and Fidell [67], we considered standardized factor loadings (*ß*) less than .55 (i.e., “good”) to be indicative of a poorly performing item requiring further examination. We first tested the measurement models for each separate construct, as specified from previous analysis of these measures, and subsequently allowed modifications based on resulting model modification indices and theoretical justification. Finally, we assessed an overall OIC model with all three constructs to examine correlations among the factors.

## Results

### Summary statistics

OIC scale and subscale item means, standard deviations, and coefficient alphas are shown in Table 2. Descriptive statistics for the 12-item ILS indicated that the Supportive subscale had the highest mean, while the Proactive subscale had the lowest mean and most dispersion. When examining the 18-item ICS, the Focus subscale had the highest mean and the Rewards subscale had the lowest mean and most dispersion. Measures of central tendency and dispersion for the 6-item ICBS revealed that the Helping Others subscale had a higher mean and lower dispersion than the Keeping Informed subscale. Skewness, kurtosis, and normality also were assessed by examining statistics and graph data of the response distributions for each of the measures and subscales. Inspection of these data indicated that all but one of the subscales had relatively normally distributed data and no significant skewness and kurtosis. The Rewards subscale from the ICS was associated with a significant positively skewed and leptokurtotic distribution. With regard to coefficient alphas, the overall ILS, ICS, and ICBS showed excellent internal consistency (i.e., > .90) most subscales in the excellent range and ICS Focus, Education support, and Recognition subscales in the good range (i.e., > .85), with only one of the 11 examined scales (Rewards from ICS––see below) yielding an alpha below .80.

**Table 2:** Measure, subscale, and item means, standard deviations, and coefficient alphas

Measure and subscales	Mean	SD	Alpha
Implementation Leadership Scale	23.99	12.15	.990
Proactive	5.33	3.13	.944
ILS1. Administrator developed a plan to facilitate implementation of EBP	1.89	1.11	
ILS2. Administrator removed obstacles to the implementation of EBP	1.71	1.02	
ILS3. Administrator has established clear department standards for EBP	1.74	1.14	
Knowledgeable	5.71	3.46	.967
ILS4. Administrator is knowledgeable about EBP	2.03	1.10	
ILS5. Administrator is able to answer my questions about EBP	1.77	1.20	
ILS6. Administrator knows what he or she is talking about when it comes to EBP	1.92	1.25	
Supportive	7.21	3.26	.956
ILS7. Administrator recognizes employee efforts to successful implementation of EBP	2.29	1.19	
ILS8. Administrator supports employee efforts to learn more about EBP	2.49	1.10	
ILS9. Administrator supports employee efforts to use EBP	2.45	1.08	
Perseverant	5.96	3.33	.968
ILS10. Administrator perseveres through the ups and downs of implementing EBP	2.06	1.16	
ILS11. Administrator carries on through the challenges of implementing EBP	2.00	1.13	
ILS12. Administrator reacts to critical EBP issues by openly addressing the problem(s)	1.92	1.16	
Implementation Climate Scale	34.99	14.54	.925
Focus	7.63	3.09	.878
ICS1. One of this school’s main goals is to use EBPs effectively	2.43	1.21	
ICS2. People in this school think that the implementation of EBPs is important	2.50	1.06	
ICS3. Using EBP is a top priority in this district	2.72	1.18	
Education support	6.53	3.16	.893
ICS4. This school provides conferences, workshops, or seminars focusing on EBPs	2.21	1.19	
ICS5. This school provides EBP trainings or in-services	2.41	1.09	
ICS6. This school provides EBP training materials, journals, etc.	1.90	1.20	
Recognition	6.18	3.32	.854
ICS7. School staff who use EBPs are seen as experts	2.32	1.21	
ICS8. School staff who use EBPs are held in high esteem in this school	2.29	1.21	
ICS9. School staff who use EBPs are more likely to be promoted	1.65	1.32	
Selection	6.39	3.44	.966
ICS13. This school actively recruits staff who have previously used EBP	2.06	1.14	
ICS14. This school actively recruits staff who have had education supporting EBP	2.12	1.19	
ICS15. This school actively recruits staff who value EBP	2.22	1.21	
Openness	7.25	3.16	.916
ICS16. This school selects staff who are adaptable	2.47	1.09	
ICS17. This school selects staff who are flexible	2.51	1.10	
ICS18. This school selects staff open to EBP	2.30	1.21	
Implementation Citizenship Behavior Scale	11.32	5.94	.989
Helping Others	5.89	2.99	.945
ICBS1. School staff assist others to make sure they implement EBPs properly	1.93	1.01	
ICBS2. School staff help teach EBP implementation procedures to new team members	1.96	1.08	
ICBS3. School staff help others with responsibilities related to EBPs	2.00	1.04	
Keeping Informed	5.45	3.24	.939
ICBS4. School staff keep informed of changes in EBPs	1.90	1.13	
ICBS5. School staff keep up with the latest news regarding EBPs	1.81	1.13	
ICBS6. School staff keep up with school communications related to EBPs	1.84	1.10	

### Confirmatory factor analyses

#### ILS

We assessed the ILS as a hierarchical CFA with items loading on the four theorized first-order factors that, in turn, loaded on a second-order Implementation Leadership factor. Fit statistics for this model were *χ*^*2*^ (50, *N* = 161) = 126.33, *p* < .01, CFI = .997, TLI = .996, RMSEA = .097 (90% confidence interval = .076, .119). All standardized factors loadings were significant (*p* < .05) and large (*ß*s > .925). Examination of modification indices suggested that model fit could be improved (∆*χ*^*2*^ = 32.93) by correlating residual error terms between two items: “*Our school administrator supports employee efforts to learn more about evidence-based practice*” and “*Our school administrator supports employee efforts to use evidence-based practice*.” However, we rejected this modification based on the rationale that the residual correlation reflected a method-related artifact arising from the common wording of the items and not from an additional implementation-related source of covariation. Fit for the modified model was *χ*^*2*^ (49, *N* = 161) = 95.13, *p* < .01, CFI = .998, TLI = .997, RMSEA = .076 (90% confidence interval = .053, .099). The correlation between the two residual error terms was *r* = .616.

#### ICS

Hierarchical CFA for the ICS construct consisted of six first-order factors of three items each, and one second-order Implementation Climate factor. Fit for model was: *χ*^*2*^ (129, *N* = 161) = 400.56, *p* < .01, CFI = .985, TLI = .983, RMSEA = .105 (90% confidence interval = .093, .116). Examination of standardized factor loadings for first-order factors indicated strong loadings for all indicators (all *ß*s > .917); however, one item, “*School staff who use evidence-based practices are more likely to accumulate compensated time”* exhibited a Heywood case (standardized factor loading >1.0) and, from inspection of model modification indices, substantial residual correlations with all other first-order factors (∆*χ*^*2*^s between 105.57 and 151.41). Deleting this one item did not substantially improve model fit; *χ*^*2*^ (113, *N* = 161) = 334.53, *p* < .01, CFI = .987, TLI = .985, RMSEA = .101 (90% confidence interval = .089, .114); and resulted in a degraded Rewards scale (i.e., *ß*s < .50), prompting deletion of this scale from the hierarchical CFA. Fit for the resulting model without the Rewards factor was acceptable: *χ*^*2*^ (85, *N* = 192) = 243.43, *p* < .01, CFI = .991, TLI = .989, RMSEA = .099 (90% confidence interval = .084, .113). Among first-order factors, one item (*School staff who use evidence-based practices are more likely to be promoted*) had a standardized loading of *ß* = .684; otherwise, all standardized factor loadings were strong (*ß*s > .817). Standardized factor loadings for the second-order ICS factor ranged from *ß* = .800 for Education Support to *ß* = .937 for Selection for Evidence-Based Programs. Although inspection of modification indices suggested that a substantial residual correlation existed between Focus and Education Support factors (∆*χ*^*2*^ = 61.50), and between two items on the Selection for Openness scale, “*This school selects staff who are adaptable*” and “*This school selects staff who are flexible*” (∆*χ*^*2*^ = 57.82), neither modification was accepted into the final model.

#### ICBS

Examination of the ICBS construct as a hierarchical CFA was not possible given that a measurement model with six items, two first-order factors, and one second-order factor is mathematically under-identified. Alternatively, we examined the ICBS as a two- first-order factor correlated (*Helping Others* and *Keeping Informed*), each with three indicators. Fit statistics for this model were: *χ*^*2*^ (8, *N* = 176) = 37.78, *p* < .01, CFI = .999, TLI = .998, RMSEA = .145 (90% confidence interval = .101, .194. All standardized factors loadings were significant and large (all *ß*s > .897). The correlation between the two factors was *r* = .887. For comparison, we also tested a one-factor model consisting of all six ICBS items as indicators. Results for this model were: *χ*^*2*^ (9, *N* = 176) = 138.99, *p* < .01, CFI = .994, TLI = .991, RMSEA = .286 (90% confidence interval = .246, .329). As these models are not nested, a test of significant difference in model fit is not available; however, the observed decrement in fit for the one-factor model, combined with our preference to adhere to the original measurement model of the ICBS, prompted us to retain the correlated two first-order factor model. No modifications to the model were warranted by modification indices.

#### OIC model

**Fig. 1:**
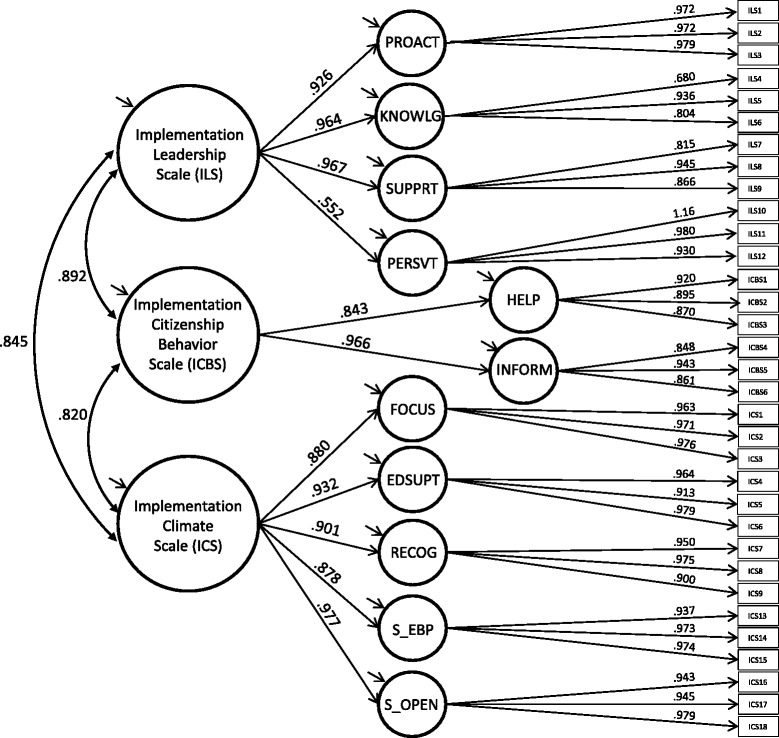
Full confirmatory factor analysis model including measures of implementation leadership, climate, and citizenship

Correlations among the 11 retained first-order factors are shown in Table 3. The three instrument-specific models were combined into a final hierarchical CFA with correlations among ILS, ICS, and ICBS factors (see Fig. 1). Fit or this model was *χ*^*2*^ (481, *N* = 193) = 1285.26, *p* < .01, CFI = .973, TLI = .971, RMSEA = .093 (90% confidence interval = .087, .099). One standardized factor loading for an item on the Perseverant Leadership Scale, “*Our school administrator perseveres through the ups and downs of implementing evidence-based practice*,” exhibited a Heywood case (*ß* = 1.15); otherwise, all standardized factor loadings for the first-order factors were within range, statistically significant (*p* < .05), and generally large (*M* = .931). Loadings for the second-order factors also were generally large, with the exception of the loading for Perseverant Leadership, which had *ß* = .552. Correlations among the second-order factors were *r* = .845 for ILS and ICS, *r* = .892 for ILS and ICBS, and *r* = .820 for ICS and ICBS. Residual variances for all factors were significant (*p* < .05) except for Keeping Informed and Selection for Openness. Inspection of modification indices showed that overall model fit could have been improved through a cross-loading of the Selection for Openness item, “*This school selects staff who are adaptable*,” on the Recognition subscale (∆*χ*^*2*^ = 220.96).

**Table 3:** Inter-factor correlations

Factor	2	3	4	5	6	7	8	9	10	11
1. Proactive	.611	.685	.539	.627	.678	.673	.575	.649	.648	.692
2. Knowledgeable		.533	.411	.463	.473	.503	.420	.470	.443	.478
3. Supportive			.515	.568	.623	.579	.569	.573	.529	.567
4. Perseverant				.529	.597	.491	.468	.481	.343	.350
5. Focus					.805	.540	.758	.741	.514	.519
6. Education support						.648	.758	.781	.605	.591
7. Recognition							.564	.817	.583	.623
8. Selection for EBP								.741	.504	.539
9. Selection for openness									.535	.582
10. Helping others										.636
11. Keeping informed										

All correlations were statistically significant at *p* < .05

*EBP* evidence-based programs

## Discussion

The primary objectives of the current study were to (1) evaluate the factor structure of revised versions of the ILS, ICS, and ICBS following their tailoring to the education sector and (2) examine their inter-relationships. The revised versions of the instruments reflect the first application of these strategic inner setting constructs to the organizational implementation context of schools. In general, the factor structures proposed and validated in the child welfare and specialty mental health sectors by the original authors were confirmed in the current analyses, with the one exception involving the removal of the Rewards subscale from the ICS (see following section for further discussion). Correlations among the measures, evaluated as part of the overarching OIC model, indicated both unique and shared variance. In the next section, we discuss the implications of these findings for future research examining implementation leadership, climate, and citizenship behavior in school-based behavioral health.

### Implementation leadership

The adapted version of the ILS appeared to function well with the current data gathered from professionals working in the education sector, supporting the relevance of the Proactive, Knowledgeable, Supportive, and Perseverant constructs in schools. Of these, Supportive leadership yielded the highest ratings, consistent with administrations of the measure in other contexts [15]. A substantial literature has previously explored principal leadership and its relationship to school climate and student educational success [68], but the development of a measure of strategic implementation leadership in schools has the potential to produce more actionable information for promoting specific leader behaviors as they relate to EBP implementation. That is, the ILS outlines specific leader behaviors that can be used to support implementation in school settings. As such, the school-specific ILS potentially could be used as a cornerstone in the application of leader-focused implementation strategies. If used at the beginning of a school-based implementation effort, the ILS could identify a need for additional leadership training, coaching, and supports prior to initiating the active implementation phase [69]. Such supports could be based on existing principal-focused leadership enhancement interventions (e.g., Coaching for Improved Leadership; [70]), which could be tailored to support implementation leadership behaviors (e.g., helping principals learn to develop plans to facilitate EBP implementation). Alternatively, recently developed implementation-specific leadership interventions–such as the Leadership and Organizational Change for Implementation (LOCI; [27, 69])–could be identified and subsequently tailored for delivery in schools.

Regardless, because many organizational theories identify leadership as a critical first step in system change–which impacts organizational climate and, subsequently, the individuals working in a given setting [14, 15, 18]–it may be that particular priority should be placed on leveraging the ILS to support leadership change in schools. As work on strategic implementation leadership in the education sector progresses, it also will be important to examine the degree to which strategic leadership constructs interact with more general leadership characteristics–such as kindness, support, and ethical leadership [71]–to impact EBP implementation. It may be predicted that leaders who possess generally positive leadership qualities and engage in strategic implementation leadership behaviors are most able to facilitate EBP adoption, delivery, and sustainment.

### Implementation climate

Decades of research has evaluated the role of molar school climate and linked it to student engagement and achievement [72, 73], but the development of a measure of a focused implementation climate in schools reflects a novel departure from this tradition. Results of the current study indicated that Focus on EBP and Selection for Openness were the highest-rated constructs in the settings assessed, suggesting that these general processes were more common than specific policies surrounding Recognition or Educational Supports for EBP.

CFA results yielded one substantial change to the school version of the ICS. Specifically, results indicated that the Rewards subscale of the ICS was not functioning adequately. As a result, the subscale was eliminated in the final school ICS model. Items on Rewards focus primarily on financial incentives and promotion for individual staff members who use EBP. Although this type of organizational support for implementation is observed relatively infrequently across contexts, it is particularly rare in the education sector [74]. School-level financial incentives for performance have been trialed [75], but individual-level tangible rewards for staff are used infrequently due to resource and policy constraints. Because its removal resulted in acceptable model fit for the ICS, and because it was generally seen as incompatible with standard practice in schools, the Rewards subscale was not retained in the final ICS model. Without the Rewards subscale, the revised ICS factor structure demonstrated a good fit to the data. Future research should not necessarily abandon the concept of rewards entirely, but seek to develop items that reflect the kinds of tangible rewards that are more likely to occur within the school context (e.g., the accumulation of discretionary or prep time).

Given that a growing body of research has emphasized the role of organizational climate for the success of implementation efforts [1, 76], a school-specific version of the ICS may be useful to promote the adoption, fidelity, and sustained use of behavioral health programming in schools. Similar to the ILS, the subscales within the ICS could be used to inform data-driven decisions across different stages of the implementation process (Exploration, Preparation, Implementation, and Sustainment) to identify specific factors (e.g., processes for staff recognition) that could be targeted via implementation strategies. Future research should examine the relationship between strategic implementation climate and molar school climate, as well as their differential influences on implementation outcomes in the education sector.

### Implementation citizenship

The current study was the first to extend the concept of implementation citizenship behavior to implementation of behavioral health interventions in schools. The adapted version of the ICBS functioned well, with the Helping Others and Keeping Informed subscales both retained in the final model and good overall model fit. Results of the instrument administration indicated slightly higher ratings for Helping Others than for Keeping Informed. Both of these subscales have considerable relevance to schools, where professional learning communities (a.k.a., learning collaboratives) are a popular strategy to support educators in improving their practice through collaborative teams of learners [77, 78]. Moreover, schools are inherently social contexts in which champions and key opinion leaders who go above and beyond the typical call of duty can have an impact on the attitudes and behaviors of coworkers to facilitate change [79, 80].

Although the ICBS is quite parsimonious, future research might consider potential expansions to the citizenship construct in schools to improve its ability to evaluate the construct more comprehensively. For instance, given that going “above and beyond” standard work obligations is the cornerstone of citizenship behavior, items assessing the extent to which staff take sufficient initiative to participate in voluntary activities related to an implementation effort (e.g., extra meetings/trainings, being observed by fellow teachers when it is not a requirement) or capitalize on opportunities to advocate for change by collectively working together to implement novel practices could be developed. Still, because the ultimate goal is to maintain a set of pragmatic measures that are useful to implementation researchers, intermediaries, and champions within the system, generation of new items or subscales for the school-specific ICBS should be done so sparingly.

### Interrelationships among scales

Although leadership, climate, and citizenship have been explored separately, little research has evaluated the constructs simultaneously. To date, none of this research has been conducted in schools. We evaluated an overarching OIC model that included all three measures as a method of examining the relationships among the instruments. Consistent with the idea that the three instruments reflect components of a larger OIC, results suggest a combination of unique contributions and shared information across the scales (Table 3). This study did not examine an exhaustive list of OIC factors, but the findings provide support for the OIC as a construct reflecting a combination of inner setting characteristics that are most proximal to and influential on EBP implementation. Future research should attempt to replicate recent path models indicating that the impact of implementation leadership on front-line professional staff is mediated by changes in implementation climate [20].

### Limitations/directions for future research

Data were only gathered from embedded behavioral health consultants within schools in the USA. As intermediaries and local champions, these individuals tend to sit at the center of school-based behavioral health implementation efforts. Still, they represent only one role in a school building among the multiple professionals who might be involved in implementation efforts [39]. Data gathered from multiple providers within each of the schools–or from multiple individuals across different professional roles–and aggregated to reflect the OIC factors at the school level might have produced somewhat different results. Given that the structure of educational systems can vary internationally, the generalizability of the current findings and revised measures beyond the context of the USA also should be evaluated. Nevertheless, this was an initial confirmatory study to examine whether the factor structure of existing OIC measures generalize to the school context. Furthermore, ILS, ICS, and ICBS scores were not directly linked to implementation [81] or service recipient outcomes. Future research may examine the degree to which the measures predict implementation outcomes (e.g., fidelity, reach, appropriateness). This study also relied on experts to revise the instruments and did not examine potential respondents’ perceptions of the relevance of the measure constructs and corresponding items for use within the educational sector. Data gathered from intended respondents could be used to inform strategic revisions to the measures to potentially further enhance their acceptability, appropriateness, and usability within schools. Furthermore, sample size was somewhat small for the overall factor analyses that included all three measures; however, sample sizes for individual-factor CFAs were within guidelines identified in the literature [82]. Nonetheless, future studies should examine the factor structure and psychometrics of these scales with larger samples. Finally, all three measures were evaluated in the same sample. Although this allowed for comparisons among the measures, it also carries a potential risk for model over-specification. Therefore, future research should confirm the models presented in independent samples.

## Conclusions

This paper reports on an effort to adapt and validate three leading measures (ILS, ICS, ICBS) capturing different facets of the OIC as they are manifested surrounding the implementation of behavioral health programming in schools. The current study benefited from an existing foundation of carefully developed, reliable, valid, and theoretically coherent measures assessing strategic implementation climate, strategic implementation leadership, and implementation citizenship behavior. All but one of the subscales from the adapted measures functioned adequately. The one that functioned poorly–the ICS Rewards subscale–appeared to do so for predictable reasons related to known constraints associated with the school context.

Despite success demonstrating construct validity in the current study, future applications or adaptations of the instruments may be necessary. Among these, adaptations could include expansion of the leadership, climate, or citizenship constructs to incorporate sub-constructs that are particularly relevant to schools. For instance, given the emphasis in schools on “response-to-intervention” and other data-driven approaches to applying academic interventions to student problems [83], it may be that the use of data to support EBP implementation reflects an important component of implementation climate in the education sector.

Furthermore, administration of each of the measures to different types of school-based personnel–especially those for whom behavioral health programming is not a central part of their professional role (e.g., teachers, administrators)–may reflect a worthwhile future direction. Teachers are the most common deliverers of universal behavioral prevention programs in schools but might have different perspectives on the OIC from dedicated behavioral health consultants. Overall, this suite of pragmatic, reliable, and structurally valid measures is likely to support high-quality implementation research and practice in schools. Subsequent development of data-driven “assessment-to-action” frameworks that link the ILS, ICS, and ICBS to specific, multi-level implementation strategies [84, 85] may represent a next frontier for the implementation behavioral health interventions in the education sector.

## Abbreviations

EBP: Evidence-based practice
ICBS: Implementation Citizenship Behavior Scale
ICS: Implementation Climate Scale
ILS: Implementation Leadership Scale
